# Waste Foundry Sand in Concrete Production Instead of Natural River Sand: A Review

**DOI:** 10.3390/ma15072365

**Published:** 2022-03-23

**Authors:** Jawad Ahmad, Zhiguang Zhou, Rebeca Martínez-García, Nikolai Ivanovich Vatin, Jesús de-Prado-Gil, Mohammed A. El-Shorbagy

**Affiliations:** 1 Department of Disaster Mitigation of Structures, Tongji University, Shanghai 200092, China; jawadcivil13@scetwah.edu.pk; 2 Department of Mining Technology, Topography, and Structures, Campus de Vegazana s/n, University of León, 24071 León, Spain; rmartg@unileon.es (R.M.-G.); jesusdepradogil@unileon.es (J.d.-P.-G.); 3 Peter the Great Saint Petersburg Polytechnic University, 195251 Saint Petersburg, Russia; vatin@mail.ru; 4 Department of Mathematics, College of Science and Humanities in Al-Kharj, Prince Sattam Bin Abdulaziz University, Al-Kharj 11942, Saudi Arabia; ma.hassan@psau.edu.sa

**Keywords:** waste foundry sand, flowability, mechanical strength, durability performance

## Abstract

The by-product of the foundry industry is waste foundry sand (WFS). The use of WFS in building materials will safeguard the ecosystem and environmental assets while also durable construction. The use of industrial waste in concrete offsets a shortage of environmental sources, solves the waste dumping trouble and provides another method of protecting the environment. Several researchers have investigated the suitability of WFS in concrete production instead of natural river sand in the last few decades to discover a way out of the trouble of WFS in the foundry region and accomplish its recycling in concrete production. However, a lack of knowledge about the progress of WFS in concrete production is observed and compressive review is required. The current paper examines several properties, such as the physical and chemical composition of WFS, fresh properties, mechanical and durability performance of concrete with partially substituting WFS. The findings from various studies show that replacing WFS up to 30% enhanced the durability and mechanical strength of concrete to some extent, but at the same time reduced the workability of fresh concrete as the replacement level of WFS increased. In addition, this review recommended pozzolanic material or fibre reinforcement in combination with WFS for future research.

## 1. Introduction

Concrete is a commonly used building raw material on the planet, and it serves as the foundation for all construction and development projects [1]. To varying degrees, each of the main constituents of concrete has an ecological influence. Because concrete is used in such large quantities worldwide, it raises a variety of sustainability concerns. A larger quantity of riverbed sand and gravels, which are concrete components, is causing increasing concern. The larger quantity removal of natural sand from the riverbed has resulted from the widespread use of concrete due to the boom in urbanization and industrialization. Enhanced riverbed distance, decreasing of the water table, revealing of bridge substructures; most importantly the influence on rivers, deltas, coasts, in addition, marine ecologies, land loss due to the river or coastal erosion, and a reduction in the quantity of deposit source are just a few of the negative consequences [2]. Furthermore, the construction industry’s survival has been severely harmed due to restrictions on sand withdrawal from the river, which has resulted in a rise in sand charges [3].

Fine aggregate (sand) is one of the essential components in producing mortar and concrete, and it plays a critical role in the design mix [4]. Sand is a main constituent of concrete, and the quantity and variety of fine aggregate used to formulate concrete will determine the properties of a specific concrete mix. It has a big impact on concrete’s flowability, resistance to environmental impacts, strength, and dry shrinkage. Sand makes up a larger percentage of the mix than cement. Sand can fill in the pores or voids in concrete, which is another factor that contributes to its strength. Sand reduces volume changes caused by the setting and hardening processes and provides a mass of particles that can withstand the action of applied loads and last longer than cement paste alone. As a result, sand plays a critical role in concrete’s ability to solidify and provide the required strength. A different alternative sand option is available to be used in concrete instead of natural sand, such as chromium sand, zirconium oxide and waste foundry sand (WFS).

Zirconium oxide is a white crystalline oxide of zirconium, with a monoclinic crystalline structure, is the mineral baddeleyite. Zirconia materials have been considered limited in their use due to their poor bonding strength to different types of cement. To enable a good mechanical bond to be established, it is necessary to have a rough surface. This is a common technique for silica-containing glass ceramics that uses hydrofluoric acid as an etching agent. However, zirconia ceramics are not reported to benefit from this method because it does not produce a surface rough enough to improve adhesion between cement and zirconia ceramics [5].

Chromite-rich sand is a source of chromite, a mineral, from which chromium metal is obtained. Chromium is primarily used as a coating on metal—termed chrome—deposited electrochemically, to protect it from rust and for decorative purposes. Different studies focus to used chromium sand in cement concrete production. According to research, A small amount of chromium has been introduced into the cement paste to accelerate hydration [6]. In a study, trivalent chromium was found to reduce total porosity and the volume of air pores in cement pastes, but not capillary pores [7]. Similar also various waste has creditability to use in concrete. The study shows that the slump value decreases with increasing chromite waste. Additionally, the results show that the addition of 5% chromite waste did not adversely affect the strength of concrete [8].

Waste production has increased as a result of rising population and technological advancements. As a result, numerous scholars and researchers around the globe are working to find innovative approaches to decrease this waste or, as a clearer option turn them into valuable reserves [4,9,10,11,12,13]. For several decades, various industrial wastes have been extensively studied as a replacement/alternative material for fine aggregate. Other materials have been discovered to enhance both mechanical and durability aspects of concrete, and this procedure can run to long-term concrete production. Different industrial waste is available to be used in concrete products, such as fly ash [14,15], silica fume [16,17], waste glass [18], and waste marble [19]. The usage of garbage items in concrete made it less expensive, and waste reutilization is thought to be the most environmentally friendly alternative for dealing with the problem of waste dumping [20]. Among this waste, WFS can be used in concrete instead of natural river sand [21].

### Waste Foundry Sand (WFS)

In foundry processes, foundry sand refers to clean, uniformly sized, high quality silica sand that is used in the casting process. Sand is bonded to form molds or patterns which are used to make ferrous (iron), non-ferrous (copper, aluminum, brass) metal castings. Components with easy or complicated structures can be created from several matters that can be molded in a single step. The foundry industry is one in which different iron, leftover steel, and ferroalloys are molten in kilns or cupolas, made in sand, ceramic, or metal molds, and produced steel, nodular, and hardened foundry goods are manufactured as raw or processed materials [22]. WFS is molding sand popular in foundry production because of its ease of use, economical, temperature resistance, and ability to bond with other binders and organic materials. In terms of performance, this sand far outperforms natural sand. This type of silt is frequently used during the production process. Whenever this sand is no longer relevant inside the production process, this is excluded and is regarded as waste foundry sand (WFS). Waste foundry sand is also recognized as used foundry sand (UFS) or spent foundry sand (SFS) [23]. UFS’s small particulates are enough too. The type of material to be poured defines the physical and chemical properties of the metal to be injected. These characteristics can vary quite a bit from one foundry to another. Approximately 100 million tons of UFS is annually generated worldwide by the foundry industry. About 1 ton of foundry sand for each ton of iron or steel casting produced is used [24]. Typically, suppliers of the automotive industry and its parts are the major generators of foundry sand (about 95% of the estimated UFS). According to EC regulations [25], UFS is classified as non-hazardous waste also because even if the total metal concentrations in waste sands are increased with respect to virgin sand, it remains generally low [26].

The two categories of foundry sand accessible are clay-bonded (Greensand) and chemically bonded sand. Greensand is perhaps the most widely used moulding medium, consisting of the aggregate, bonded with a mixture of mainly clay and water which as Chemically Bonded sand moulds are created using a wood, metal, or plastic pattern. Steel casting foundries in the United States unload roughly nine million tons of expended sand into garbage dumps every year [27]. Foundry sand is used to mould foundry products in places, such as factories and workshops that produce parts for the automotive, construction, machine industries, and the steel industry (iron-steel industry and aluminium- and copper-based alloys). Sand moulds are used in a large part of the foundry process. Metal foundry moulds are prepared with foundry sand; 4–5 tons of sand are required for one ton of foundry. This ratio may be altered depending on the type of metal to be cast, the size of the part, and the moulding technique used. Foundry sands are sands that have a sintering temperature of over 1500 °C and contain more than 90% silica and 7–15% clay (bentonite or kaolinite clay). Foundry sands can be found in abundance in nature [28]. According to industry estimates, almost 100 × 10^6^ tons of foundry sand are used in production each year; about four to seven million tons are wasted each year and offered for recycling [29]. The fast growth in industrialization, specifically foundries, has led to an accumulation of WFS which is considered an environmental concern. In foundries, the main raw material used is greensand, which is used repeatedly during moulding [30]. Sand becomes unusable after several mouldings, cooling, and recycling processes, so it is stored as waste [31]. Currently, less than 30% of the million tons of WFS that are generated each year are recycled and the remainder is stored outside foundries where storage space is limited [32]. In the United States, the amount of waste from foundries ranges from 6 to 10 million tons annually [33].

**Figure 1:**
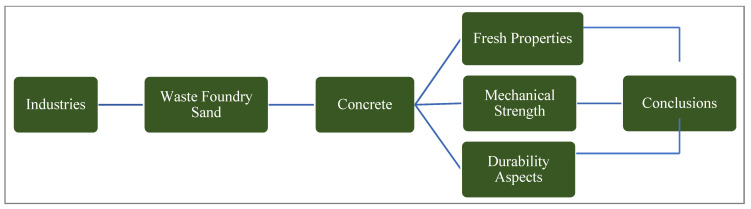
Flow chart.

One of the major issues faced by foundries is the accumulative capacity and management of the stockpiled WFS as it requires a large space for accumulation. There is a risk associated with stockpiled waste foundry sand due to the high rate of toxic metal leaching from the waste foundry sand, which results in WFS posing a threat to the sustainability of the environment [34]. Hence, foundries must develop innovative solutions to reutilize the stockpiled waste foundry sand to adopt circular economy concepts before the stockpiled WFS exceeds the storage capacity, which could slow down production [35]. In addition, the strict dumping regulations and rules are designed to reinforce the industries’ efforts and commitments to improve their waste re-utilization or recycling rates to support the zero-waste goal. Currently, landfilling is not considered to be the best alternative and is discouraged in a world where concepts, such as circular economy, enhancing environmental sustainability are emphasized to reach the zero-waste goal [36]. Several foundries are contending with high disposal penalties as a result of landfilling, which has become a financial burden that is not sustainable and affects their profit margins [37]. This paper reviews the suitability of WFS in concrete production instead of natural river sand. Figure 1 shows the flow chart of the review.

## 2. Physical Properties

**Figure 2:**
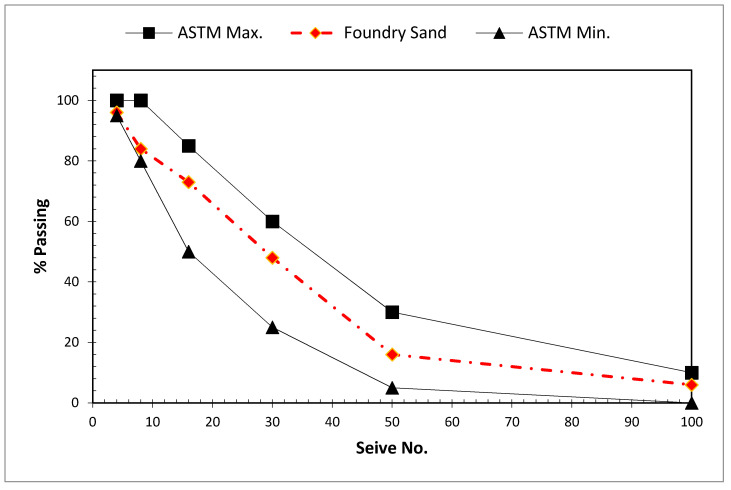
Particle Size Distribution of WFS [39].

The physical properties of industrial wastes, including grain size distribution, density, specific gravity, fine substance, and absorption, assist in determining their applicability and ability to utilize fine aggregate in concrete. WFS is usually semi-circular or circular in shape. It has a consistent grain size distribution, with 85–95% of it having grains between 0.6 and 0.15 mm and 5–12% having grains smaller than 0.075 mm [38]. Figure 2 shows particle size distribution outcomes of WFS as reported by Ahmad et al. [39] with regard to ASTM C33 [40] higher and lower limits for fine aggregate. The specific gravity of WFS was observed to be between 2.4 and 2.60, approximately equal to natural sand (2.65). The fineness modulus of WFS was noticed to be 1.78, which is lower than the 2.3–3.1 found in normal sand. The average unit weight of WFS was 1600 (kg/m^3^) which is equal to the natural sand (1600 kg.m^3^). The existence of binders and additives was found to cause reported values of WFS absorption to vary greatly but are usually on the greater end of normal sand. Numerous foundries even use binders, such as clay, sawdust, and wood for molding. The existence of these elements lowers the material’s specific density while also lowering the density of the concrete by making air voids. The percentage of particles smaller than 75 microns is 18 to 24 percent. Table 1 shows lists some of the physical properties of WFSs indicated by various researchers.

## 3. Chemical Properties

According to previous research, the chemical composition of WFS is displayed in Table 2 below. In terms of chemical contribution in the WFS, SiO_2_ is significantly abundant. In comparison to SiO_2_, the ratios of the other components are extremely low. The chemical composition of WFS varies depending on the kind of metal, binder, and combustible applied, and this impacts its operation. In sands from a single foundry, on the other hand, the properties of WFS do not show the difference with time. Additionally, Blended sands manufactured by foundry consortiums commonly generate sands with the same constituents. WFS has a high silica content and resins/chemicals, bentonite, sea coal and dust [45]. A study confirms that [46] the chemical composition of WFS is according to the ASTM C 618 [47].

## 4. Fresh Properties

### 4.1. Workability

Concrete workability measures the ease in which fresh concrete could be placed, consolidated, and finished to minimum losses of uniformity. Any concrete mixture should be feasible enough to be adequately placed and consolidated and fill the forms and surround the reinforcement or other embedded items. The workability and strength of concrete are directly related to each other. The strength of concrete increases as the flowability of normal concrete increases, affecting the concrete’s durability. Workability impacts placement and finishing operations’ capacity, performance, appearance, and sometimes even labour costs. When it comes to optimum concrete design, there are a variety of expectations and agendas among the design/construction team. The structural engineer desires increased strength as well as a strong bond with reinforcing steel. The architect is involved regarding esthetics. Load-carrying capacity is valuable to the holder since it allows for relatively small cross-sections of structural members hence more serviceable floor space. A labourer demands concrete mix that can flow, place, and compact quickly and easily, while a finisher wants something that can be durable and gives better finishing. A concrete blend along with excellent flowability balances several characteristics, resulting in a high-quality product with long service life. Concrete compaction is reduced, and porosity is increased due to poor concrete workability. The concrete density decreases as porosity increases, resulting in lower compressive strength. One of the most critical aspects to consider is your ability to work.

**Figure 3:**
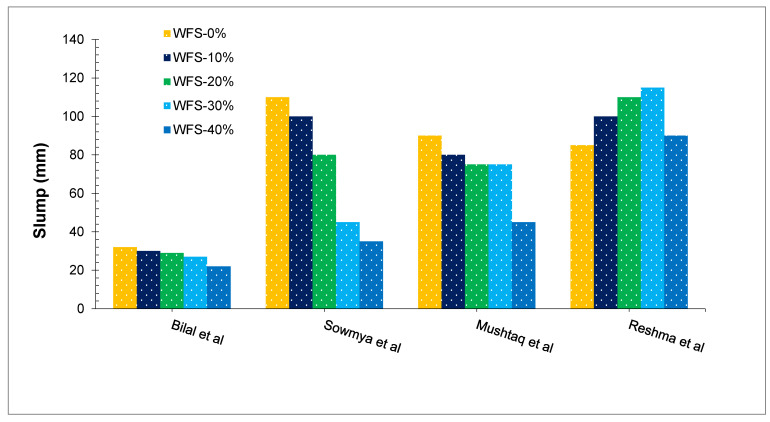
Slump with WFS: Bilal et al. [43], Sowmya et al. [50], Mushtaq et al. [51] and Reshma et al. [52].

Figure 3 shows the flowability of fresh concrete with different proportions of WFS as reported by the researchers. Generally, the flowability of fresh concrete is reduced with the substitution of WFS. The physical aspects of WFS, such as fineness and porousness, cause a decrease in the workability of concrete blended with it. On the other hand, fine particles raise the viscosity of paste due to their higher surface area, reducing the flowability of concrete. Bilal et al. [43] studied the impact of WFS on concrete performance by varying the dose from 0 to 40% in ten percent increments. The workability of concrete was found to be reduced when WFS was substituted. Beyond 30 percent replacement, a mix containing a high percentage of WFS substitution ratio becomes harsh, sticky, and stiff/inflexible. Additionally, in mixing and placing, the mix was not as harsh up to 30%. A 30 percent replacement rate resulted in a nearly 15 percent decrease in slump value. The reduction in slump value was increased to around 31% at the 40 percent replacement point. This reduction in flowability is most likely due to the existence of the water-absorbing nature of WFS (porous). Ahmad et al. [39] also stated that the flowability of concrete reduced with the replacement of WFS. The greater the water absorption and fineness of the (WFS), the greater the requirement for water in concrete, causing reduced flowability of concrete mix. The fineness of the (WFS) improves the appearance of hydration products, resulting in more water absorption. However, up to 30% substitution of WFS is acceptable regarding flowability. Despite this, several performances were observed in this study, which could be ascribed to weakening the aggregate-paste bond. Extra fine particles weaken the bond between aggregate and cement paste, resulting in decreased adhesion and improved concrete workability [48]. A study also found that increasing the partial replacement of WFS in the constant water-cement ratio of 0.44 reduced the slump value. With a substitution rate of up to 10%, they found that the effect of WFS on flowability was not considerable, and the flowability of concrete was equal to the reference concrete [49]. A summary of different properties of concrete with partially substituted WFS is presented in Table 3.

### 4.2. Compacting Factor (C.F)

**Figure 4:**
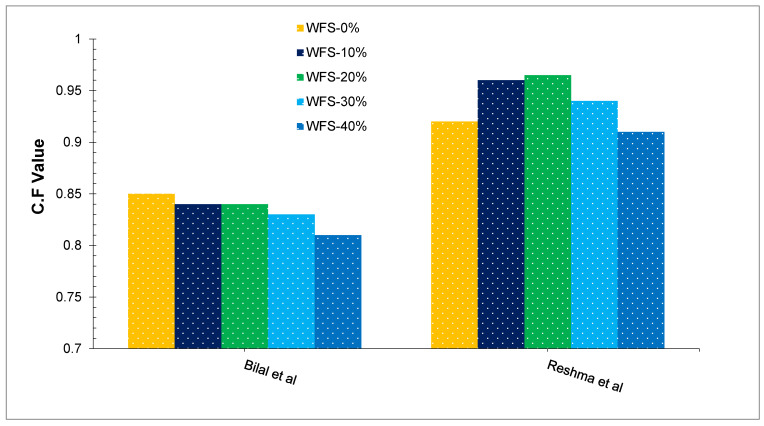
Compaction Factor: Bilal et al. [43] and Reshma et al. [52].

The compacting factor analysis is performed mainly for experimental needs, but it can also be used in the field. It is more precise and sensitive than the slump test, and it is particularly suitable for low flowability concrete mixes. It’s also frequently used when concrete is compacted with a vibrator. Bilal et al. [43] found that the compacting factor value of concrete with partially substituted WFS ranging from 0% to 40% in 10% increments lies between 0.85 and 0.81, as shown in Figure 4. A study that replaced natural sand with WFS and bottom ash with a constant compaction factor of 0.78–0.83 found that increasing sand replacement with waste foundry sand and bottom ash increased water demand [53]. Reshma et al. [52] reported that the compacting factor value of concrete lies in between 0.91 to 0.96 with partially substituting WFS [52]. Similarly, a study reported a compacting factor value of 0.90 to 0.94 [54]. It can be observed that fewer researchers reported compaction factor tests in their research.

## 5. Mechanical Properties

### 5.1. Compressive Strength

Concrete’s compressive strength is its ability to resist compressive loads before actually failing. The compression test seems to be the most essential of several concrete assessments since it gives information about the concrete’s characteristics. The concrete compression strength after 28 days curing, as ascertained by different researchers, in which fine aggregate is full or partial substitute with WFS, is displayed in Table 3.

**Figure 5:**
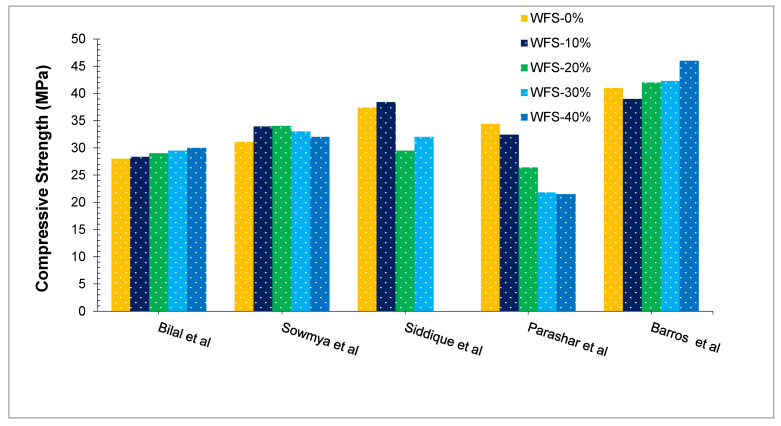
Compressive Strength at 28 days: Bilal et al. [43], Sowmya et al. [50], Siddique et al. [59], Parashar et al. [58] and Barros et al. [57].

Figure 5 shows the compressive capacity of concrete with various doses of WFS. Bilal et al. [43] conclude that the compressive strength of concrete with partially substituted WFS is increased as compared to the reference concrete. This might be due to the existence of finer grains in WFS, which acted as a tremendous filling material and led to a denser concrete mix [55]. The void-filling of granular materials reduces the number of pore spaces inside the hardened concrete, which tends to result in a tightly packed matrix [45]. The existence of silica might have helped in the creation of the calcium silicate hydrates (CSH) gel [39]. CSH is formed due to the chemical reaction of SiO_2_ present in WFS with calcium hydrate (CH) formed during the hydration of cement. CSH improves the binding aspects of concrete leading to more strength. The decrease in compressive strength was observed with further than 30% substitution of WFS. With further than a 30 percent substitution, a decrease in strength was noted. This noticeable drop in strength properties with the addition of 40% WFS might be credited to an increase in the surface area of fine particles of WFS, resulting in a reduction of the water-cement gel in the matrix. As a result, the coarse and fine aggregate binding process is flawed [45]. The results of this study in compression strength seem to be in line with the observations of many other research findings [56,57]. In contrast, Parasha et al. [58] used WFS as a partial alternative for natural river sand in concrete in proportions of 0, 10, 20, 30, and 40%. Results indicate that concrete compressive strength is decreased when WFS is substituted. Its fine and porous nature accounts for the decrease of compressive capacity when (WFS) is included. (WFS) consuming more water and having a finer particle size increases water requirement in the concrete, causing low flowability and also leading to a decrease in the concrete compacting, creating a greater number of tiny holes close to the aggregate surfaces. Materials similar to sawdust, wood flour, and clay cause a reduction in the density of the respective materials and also cause a reduction in the density of concrete by creating air spaces inside the structure [49]. For significant concrete improvement, it may be recommended that WFS is used to a maximum of 30% of its volume in order to produce economical, more environmentally friendly concrete. A similar result was observed in other research, in which natural sand was substituted with WFS at a 30% rate, and the results presented a satisfactory response. So in order to achieve optimum results, it would be advisable to replace WFS up to a 30% level with natural sand [42,59].

### 5.2. Split Tensile Strength

Concrete’s tensile strength is one of its fundamental and most significant characteristics which influences the amount and range of cracks that occur in a structure. Furthermore, the concrete is weak in tension owing to its brittle nature, which makes it prone to cracks. This means that it will not be able to withstand direct tension. Accordingly, cracks develop when tensile forces go beyond the tensile strength of concrete. As a consequence, it is required to ascertain the tensile capacity of concrete to detect the limit of load at which the concrete can crack. Additionally, the splitting tensile strength test on a concrete cylinder is a method that can be used to determine the tensile capacity of concrete. It is carried out in accordance with ASTM C496 [60].

**Figure 6:**
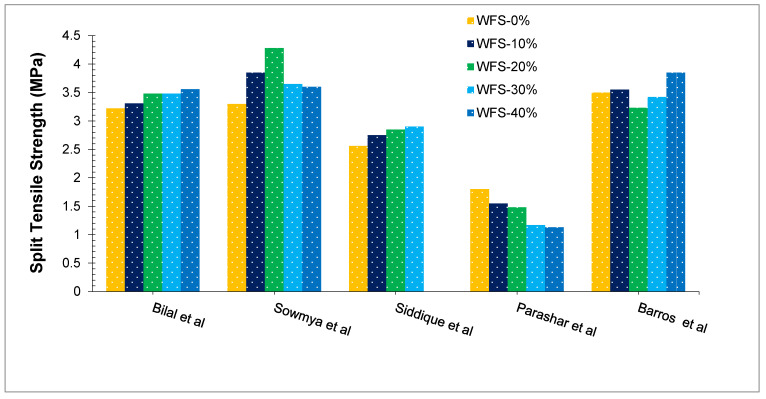
Split Tensile Strength at 28 days: Bilal et al. [43], Sowmya et al. [50], Siddique et al. [59], Parashar et al. [58] and Barros et al. [57].

Figure 6 shows the split tensile strength of concrete with various doses of WFS. Ahmad et al. [39] reported that WFS was partially substituted for natural river sand in their analysis. It is due to the porosity enhancement, which results in a lower density structure as a result of the presence of fine dust particles in the WFS [49]. Although the split tensile strength of cement concretes with up to a 30% replacement of WFS is almost the same as that of the reference mix. Prabhu et al. [61] found that concrete mixed with prewashed four and sun-dried, up to a 20% substitution ratio of WFS would be comparable to the control mix. However, after 30% replacement, a minor decline in strength was detected and further decreased at a greater dose of WFS. At a 50% substitution ratio of WFS, 19% less split tensile strength was observed as compared to the blank mix. Bilal et al. [43] conclude that, in addition to increasing the compressive strength of concrete, the split tensile strength also improved, when compared to the control concrete with partially substituted WFS. The splitting tensile strength of SCC concrete with 15% WFS content was significantly higher than the concrete without WFS [62]. In contrast, Parasha et al. [58] concluded that the replacement of WFS decreased split tensile strength. According to Sowmya et al. [50], the split tensile strength of concrete increased up to 20% substitution of WFS. Subsequent substitutions have shown a decrease in split tensile strength. A study performed by Basar et al. [63], has determined that the tensile capacity of concrete reduces systematically as the amount of waste foundry sand is substantially increased. A comparable and steady reduction in the tensile capacity of concrete with an rise the substitution ratio of WFS has been observed by the past researcher [64].

### 5.3. Flexure Strength

**Figure 7:**
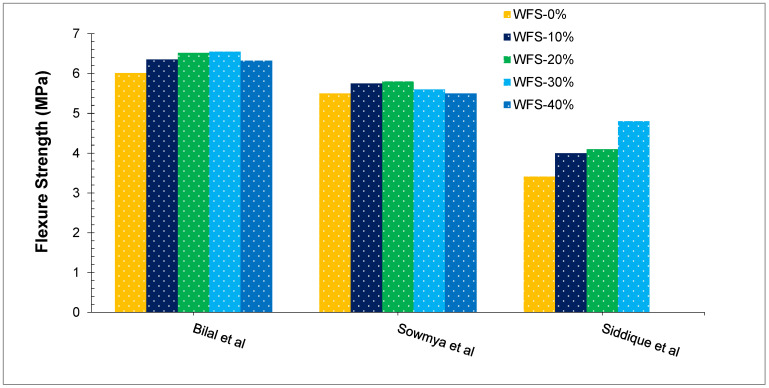
Flexure Strength at 28 days: Bilal et al. [43], Sowmya et al. [50] and Siddique et al. [59].

The tensile strength of concrete can be indirectly measured by its flexural strength. The maximum tension is applied to the tension face of an unreinforced concrete beam or slab at the point of failure during bending testing. The strength of concrete is determined by loading 150 × 150 mm (or 100 × 100 mm) concrete beams with a span length greater than three times the depth. Flexural strengths are expressed as Modulus of Rupture (MR) in MPa and are calculated by standard tests ASTM C78 [65]. Figure 7 illustrates the flexural strength of concrete according to past research with different doses of WFS in terms of flexure strength. Ahmad et al. [21] conclude that the flexure strength is also reduced with an increase in the rate of (WFS). Though no improvement in strength was observed with substitution WFS in concrete, the concrete flexure strength of blends with up to 30 percent addition of (WFS) was nearly the same as the strength of blank blend (WFS0 percent). When compared to the concrete mix without any addition of WFS, the concrete mix containing 30 percent of WFS has a 9.1 percent lower flexure strength after 28 days of age. The results from a study also showed a marginal decrease in flexure strength with the substitution of WFS to reach a higher rate [66]. Despite the differing results, Prabhu et al. [49] concluded that the flexural strength of the blends up to a 20% replacement ratio was comparable with the control concrete. Further addition of WFS leads to decreased flexure strength. Sowmya et al. [50] observed a maximum flexure of 5.8 MPa at 20% substitution of WFS. Based on the study, foundry sand content marginally increased concrete flexural strength [67]. In the 28-day evaluation period, the WFS of the control mixture M-1 (0% WFS) was found to be 6.15 MPa, whereas mixtures with 10%, 20%, 30%, and 40% of WFS showed flexure strengths of 6.34, 6.65, 6.78, and 6.27 MPa, respectively, indicating a marginal increment of 3.12%, 8.20%, 10.35%, and 2.01%, respectively, concerning the control mixture. The substitution level of 30% showed marginal increases on all the days of testing. However, the strength decreased after 30% substitution [43]. However, Thiruvenkitam et al. [32] observed a considerable improvement of flexure strength with substitution of WFS. Maximum flexure strength was seen at 15% replacement of WFS which was 12% more than reference concrete. A study also noted that a 30% substitution WFS with 10% rice husk ash shows maximum strength [68].

## 6. Durability

Concrete can be described as durable if it can withstand weathering action, chemical attack, abrasion and any other deterioration process. In adverse environments, durable concrete must maintain its original form and serviceability. The durability of concrete can be detected through various tests, such as water absorption, density, acid attacks, dry shrinkage, ultra-sonic pulse velocity, etc.

### 6.1. Water Absorption

**Figure 8:**
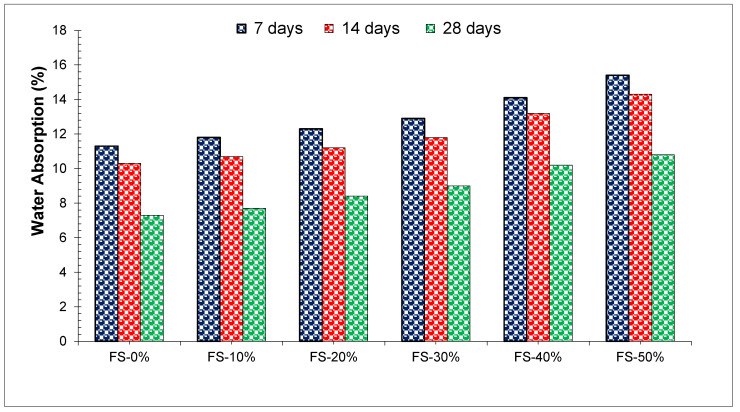
Water Absorption [39].

An assessment of concrete durability (water absorption) is an indirect measure of concrete porosity that is an indirect measurement of concrete durability. The water absorption capacity and the porosity of hardened concrete play important roles in its durability. To further increase the flexural and compressive strength and durability of concrete, it is important to reduce its porosity [75]. Figure 8 shows WA of concrete with different doses of WFS. A study reported that WA reduced with the replacement of WFS [35]. Ahmad et al. [39] found 5.4%, 5.8%, 6.4%, and 6.6% on concrete which contains 10%, 20%, 30% & 40% of waste foundry sand at 28 days of age as in comparison to control concrete mix.

Conventional concrete without waste foundry sand (WFS) results in the least amount of water consumption and there is a significant increase in water absorption rate when the rate of replacing WFS is increasing [63]. Additionally, we noticed that no apparent effect in WA was observed with the substitution of WFS. Concrete with 20% substitution WFS shows only 1.13% more WA as compared to the control concrete [75]. The replacement of WFS does not have a significant effect on water absorption up to a replacement level of 30%. According to a study, water absorption levels for concrete with 10%, 20%, 30% and 40% of WFS were 5.4%, 5.8%, 6.4%, and 6.6% at 28 days which is similar to the control concrete water absorption (5%). Traditional concrete with no WFS has the lowest water absorption ratio, while the water absorption ratio increases when WFS is substituted at higher rates. According to the study, foundry waste sand does not have an obvious effect on water absorption except for concrete with 20% ferrous waste sand that absorbs 1.13% compared to the control mix that absorbs 1.91%. According to a study, concrete with partially replaced WFS has about the same water absorption as control concrete [57]. Water absorption usually increases with WFS in concrete. Increased water absorption leads to a decrease in compressive strength [76].

### 6.2. Acid Attacks

An acid attack occurs when acid-susceptible constituents, such as calcium hydroxide and hydrogen peroxide are dissolving and leaching from the cement paste of hardened concrete. As a consequence of this action, there is an increase in capillary porosity, a decrease in cohesiveness, and ultimately reduced strength. Cracking and disintegration may occur as the result of an acid attack, particularly when the structure is exposed to water pressure on one side.

**Figure 9:**
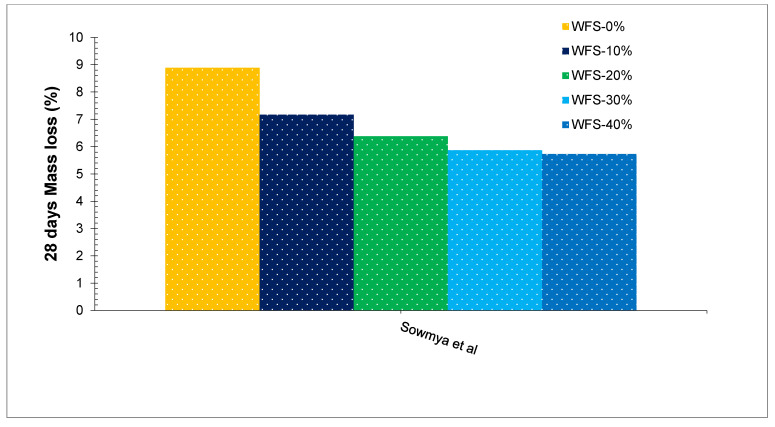
Acid Resistance at 28 days [56].

Figure 9 shows sulfuric acid attacks on concrete with varying dose WFS. It can be observed that acid attacks due to sulfuric acid decreased with the substitution of WFS instead of natural river sand. Least acid resistance was detected at 0% replacement of WFS while maximum acid resistance was detected at 40% replacement of WFS as shown in Figure 9. A study concludes that after immersion in H_2_SO_4_, concrete with WFS shows less corrosion compared to the reference concrete [77]. However, less information is available on acid attacks on concrete with the substitution of WFS.

### 6.3. Density

It is well known that concrete’s mechanical properties are greatly influenced by the density of the concrete. In general, dense concrete provides higher strength and less porosity and voids.

**Figure 10:**
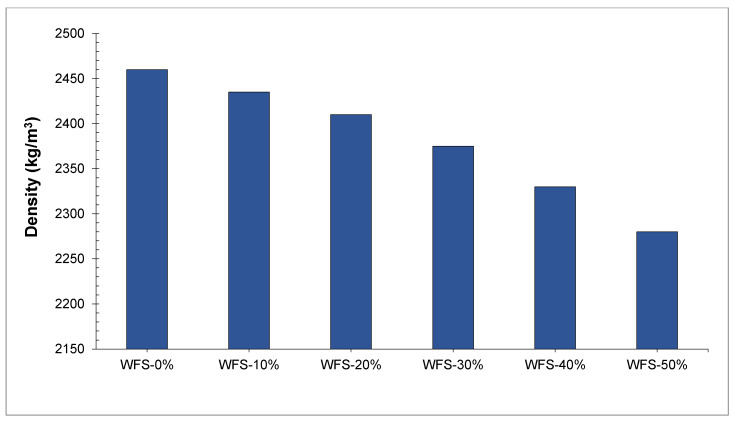
Density of concrete [39].

Figure 10 shows the density of concrete with various doses of WFS. The density of concrete was enhanced with the substitution of WFS. It is expected that the micro filling effect of WFS fills the voids in concrete ingredients leading to more dense concrete. A researcher also stated that the density of concrete improved with partial replacement of WFS due to micro filling voids of WFS [57]. In contrast, Ahmad et al. [39] stated that the density of concrete reduced with the replacement of WFS. The highest density is achieved at 0% replacements of WFS while the minimum density was achieved at 50% replacement of WFS. The reduction in density of concrete made with waste foundry sand is expected, due to the physical characteristics of waste foundry sand which are fineness and porous. The more water absorption and fineness of the waste foundry sand (WFS) enhances the water requirement of concrete due to water absorption, which leads to decreased concrete density. A study indicated that the concrete density in the hardened stage decreases as the rate of replacement of waste foundry sand (WFS) increases. In addition to that, they also stated that many foundries continue to use sawdust and clay as glues when making moulds. This decreases the specific density of the material and also reduces the density of the concrete by creating air vacuums within the concrete particles [49]. Siddique et al. [67] suggest that the fresh concrete density of the control blend is roughly the same as the concrete density obtained after waste foundry sand (WFS) was used as a replacement for common sand from ten percent to thirty percent.

### 6.4. Carbonation Depth

Concrete carbonation is one of the major factors associated with the corrosion of steel reinforcements in concrete structures. A general rise in carbon depth was observed as the replacement level of WFS increased.

**Figure 11:**
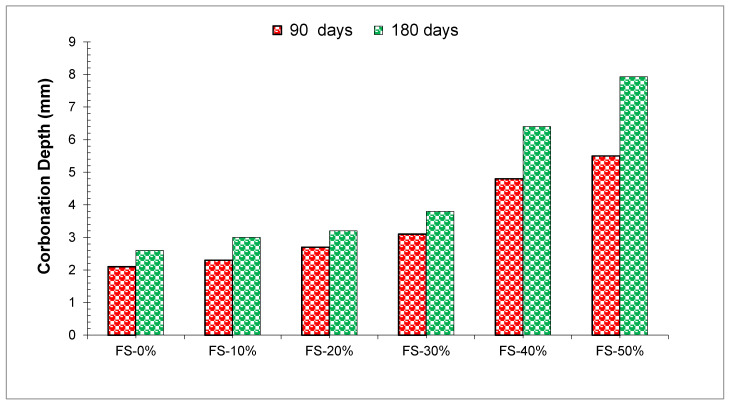
Carbonation Depth Test Results [39].

Ahmad et al. [39] stated that with the use of WFS in concrete, the chloride penetration value of concrete was enhanced and the enhancement in penetration value was directly correlated with the rate of replacement of the waste foundry sand as shown in Figure 11. Therefore, the penetration values of the compositions with a substitution ratio of (WFS) up to 30 percent were reasonably similar to the penetration values obtained for the control mixture. The penetration value of a concrete blend containing 30 percent WFS was 621 coulombs at 180 days of age, while a control concrete blend obtained a penetration value of 420 coulombs, only 32.36 percent less than that of the blend containing 30% WFS. However, the penetration value for the 30 percent of WFS is much lower than the maximum value suggested by the American Society for Testing Materials (ASTM) C1202-97 [78]. It was found that the RCPT values at 90 days were 578, 628, 616, 600, 664, 652, and 741 in concrete blends containing fine aggregates substituted with 10 percent, 20 percent, 30 percent, 40 percent, 50 percent, and 60 percent by WFS. The results of this study establish that the chloride-ion absorption properties of concrete blends were enhanced with an increase in WFS [53]. In the same way, the difference in penetration value was observed at 365 days of age. Overall, the resistance to chloride penetration is greater when more C_3_A is created in the binder, resulting in greater resistance to corrosion. The WFS used in this study contains 4.93 percent Al_2_O_3_, which is similar to cement. Even though Al_2_O_3_ and SiO_2_ can be found in the WFS to create the densified tri-calcium aluminates (C3A), the low workability of the concrete causes difficulty in the compaction process, leading to the formation of a continuous permeable microstructure. There is an additional possibility that, as a result of the WFS grains being present in the concrete, air pockets were created in the concrete. A crack or an opening in the concrete may have allowed the water to penetrate. It was because of the creation of this continuous pore system that chloride ions were able to penetrate. It appears that blends of WFS 40 percent and WFS 50 percent have a considerably greater chloride penetration compared to the control mix. Generally, the replacement of the WFS in concrete has a deep effect on the penetration of chloride. Although, this effect was not critical up to a replacement ratio of 30, the penetration value was generally accepted as being much lower at both ages of concrete. 

## 7. Conclusions

This paper examined the utilization of WFS as a fine aggregate in concrete production. In this review, all the essential properties, such as the physical and chemical composition of WFS, fresh properties, mechanical and durability performance of concrete have been discussed and compared. Even though WFS has a few harmful impacts on concrete performance when utilized in a higher or full substitution instead of natural sand in concrete production; it can be utilized in concrete production up to a certain extent. Several investigations have been conducted regarding the use of WFS in concrete. The optimum substitution dose is determined to be 30% concerning most of the properties tested. Furthermore, a detailed conclusion is listed below.

- There was almost no difference in bulk density, specific gravity, or grain size distribution between WFS and natural sand.
- Flowability of concrete reduced with the substitution of WFS. This is owing to the physical properties of WFS (porous and larger surface area) which increased water demand. However, up to 30% substitution of WFS shows acceptable workability but a higher dose (beyond 50%) needed a higher dose of admixture (plasticizer).
- WFS can be used up to 30% substitution instead of natural river sand with no harmful influence on concrete strength. This is owing to the micro filling which provides more dense concrete, leading to more resistance to load. However, a decrease in strength was observed at a higher dose of WFS (beyond 50%). The reason for the decrease in strength is the lack of workability that increases the difficulty in the compaction process, which results in more voids in the hardened concrete.
- Adding WFS tends to decrease its mechanical strength. The lack of workability caused pores to develop in concrete and less paste to be available for binding, resulting in reduced strength. Results can be comparable to the control concrete at a 20% replacement level of WFS. A 30% replacement of WFS has been suggested in some studies.
- Improvement in durability aspects (water absorption, acid resistance, density and carbonation depth) of concrete with WFS was observed. This is due to the dense matrix due to the addition of fine WFS.

Finally, this overall review concluded that WFS up 20 to 30% can be used as fine aggregate in concrete production without any negative effect on the mechanical and durability performance of concrete. Furthermore, less information is available on the durability performance of concrete with WFS. Therefore, this review strongly recommended a detailed study on the durability performance of concrete with partially substituted WFS. Additionally, up to 30% substitution of WFS shows the mechanical performance of the concrete is approximate to the control concrete. Further study was recommended to add fibers or some of the pozzolanic materials, such as fly ash or silica fume to improve the mechanical performance.

## Figures and Tables

**Table 1:** Physical properties of WFS.

Authors	Divya et al. [41]	Siddique et al. [42]	Ahmad et al. [39]	Bilal et al. [43]	Guney et al. [44]
Specific gravity	2.4	2.61	2.34	2.55	2.45
Absorption (%)	1.7	1.3	4.08	1.48	-
Fineness Modulus	1.86	1.78	2.33	1.90	-
Moisture Content (%)	Nil	-	-	-	3.25
Particle finer than 75 µm (%)	-	18	-	-	24
Unit weight (kg/m^3^)	-	1638	1546	1555	-

**Table 2:** Chemical Composition of WFS.

Authors	Ahmad et al. [39]	Divya et al. [41]	Bilal et al. [43]	Guney et al. [44]	Dogan et al. [31]
SiO_2_	81.8	88.11	88.50	98	98.64
Al_2_O_3_	6.9	0.49	4.63	0.8	0.74
Fe_2_O_3_	2.3	2.38	0.83	0.25	1.01
MgO	0.32	0.76	0.21	0.023	0.50
CaO	3.55	1.65	0.90	0.035	0.35
NaO_2_	0.6	0.95	0.02	0.04	1.07
K_2_O	0.9	0.83	0.01	0.04	0.21

**Table 3:** Summary of Fresh and Mechanical performance of concrete with WFS.

Author	WFS Replacement with Fine Aggregate	Slump (mm)	Compression Strength (MPa)	Flexure Strength (MPa)	Split Tensile Strength (MPa)
Mynuddin et al. [66]	0%		24.36	4.58	1.6
	50%		22.34	4.28	1.8
	100%		19.7	4.13	1.4
Bilal et al. [43]	0%	32	28.0	6.01,	2.5
	10%	30	28.36	6.35,	3.22
	20%	30	29.0	6.52,	3.31
	30%	27	29.5	6.55,	3.48
	40%	22.	30.0	6.32.	3.56
Raja et al. [69]	WFS–CS%				
	10–10	49	28.9	3.92	2.99
	20–10	42	30.4	4.12	2.95
	30–10	32	32.1	4.26	2.88
	10–20	41	27.5	3.86	2.79
	20–20	38	24.2	3.69	2.62
	30–20	30	22.3	3.57	2.5
Jadhav et al. [70]	0%	-	27.17	-	-
	10%		29.79		
	30%		30.66		
	50%		29.07		
	100%		25.58		
Sowmya et al. [50]	0%	110	31.11	5.5	3.3
	10%	100	33.92	5.75	3.85
	20%	80	34.04	5.8	4.24
	30%	45	33	5.6	3.65
	40%	35	32	5.55	3.6
Bhandari et al. [71]	0%	-	32.58	-	-
	10%		32.87		
	20%		33.51		
	30%		18.21		
	40%		10.74		
	60%		5.37		
	80%		3.22		
	100%		1.57		
Siddique et al. [67]	0%	-	37.4	3.41	2.56
	10%		38.4	4.00	2.75
	20%		29.5	4.10	2.85
	30%		30.5	4.18	2.90
Mavroulidou et al. [35]	0%	-	46	4.6	2.85
	10%		50.5	4.9	2.95
	30%		49	4.8	2.9
	50%		48.5	4.85	2.9
	70%		46	4.85	2.9
	100%		48.5	485	2.85
Parashar et al. [58]	0%	-	34.4	-	1.08
	10%		32.4		1.55
	20%		26.4		1.48
	30%		21.8		1.17
	40%		215		1.13
Kumar et al. [72]	Control	-	20	3.13	1.21
	QD: WFS				
	70:30		22.2	3.57	1.27
	80:20		23.3	3.78	1.38
Thiruvenkitam et al. [32]	0%	-	34.5	5.75	2.3
	5%		35	5.85	2.46
	10%		35.5	6.30	2.5
	15%		37	6.45	2.57
	20%		35.4	5.90	2.48
	25%		33.5	5.85	2.35
Reshma et al. [52]	0%	85	40.56	4.12	4.96
	10%	100	42.35	4.25	5.12
	20%	110	42.96	4.36	5.23
	30%	115	43.05	4.47	5.36
	40%	90	41.26	4.15	5.05
Kavitha et al. [73]	Treated: Untreated		-		
	0	118		38.15	4.1
	10:10	100:99		41.96:40.39	4.42:4.34
	20:20	90:89		44.15:42.25	4.65:4.57
	30:30	84:83		49.29:45.34	4.94:4.74
	40:40	80:78		43.38:41.37	4.35:4.55
	50:50	65:62		40.25:38.55	4.21:4.33
Mushtaq et al. [51]	0%	90	34	-	3.00
	10%	80	27		2.80
	20%	75	30		2.90
	30%	65	32		2.95
	40%	45	33		2.50
	50%	25	27		2.45
Zai et al. [74]	WFS: GF				
	0:0	100	42	6.24	3.07
	40:0.5	85	37	6.68	3.106
	50:0.5	75	35	6.4	3.09
	40:1	80	46	6.76	3.64
	50:1	100	37	6.48	3.17
Barros et al. [57]	0%		41		3.5
	10%		39		3.55
	20%		42		3.23
	30%		42.3		3.42
	40%		46		3.85
	50%		44		3.75
Manoharan et al. [54]	0%	110	24.0	4.84	2.2
	5%	100	24.5	4.97	2.3
	10%	100	25 25	5.06	2.4
	15%	90	25.9	5.14	2.6
	20%	85	26.3	5.2	2.8
	25%	80	22.3	4.78	2.1
Prasad et al. [41]	M: FA: WFS: PP				
	M1: 20: 10: 0.5	130	32	5	3.18
	M2: 20: 20: 1	79	36.5	5.19	3.82
	M3: 20: 30: 1.5	37	29	4.34	2.86
	M4: 25: 10: 0.5	84	30	5.78	3.50
	M5: 25: 20: 1	43	34.5	3.90	3.34
	M6: 25: 30: 1.5	119	33	4.88	3.66
	M7: 30: 10: 0.5	51	25.5	5	2.86
	M8: 30: 20: 1	124	35.5	4.98	4.13
	M9: 30: 30: 1.5	73	37	5.15	3.50
	CM: 0: 0: 0	160	34.5	4.67	2.86
Manoharan et al. [54]	0%	110	24.0	4.84	2.2
	5%	100	24.5	4.97	2.3
	10%	100	25 25	5.06	2.4
	15%	90	25.9	5.14	2.6
	20%	85	26.3	5.2	2.8
	25%	80	22.3	4.78	2.1
Siddique et al. [55]	0%	90	30		3.4
	5%	85	34		3.6
	10%	85	37		3.85
	15%	80	38.5		3.9
	20%	75	37.5		3.8

CS = Coconut Shell; QD =Quarry dust; GF = Glass Fibers; FA = Fly Ash; PP = Propylene Fibers.

## Data Availability

All Data are available in the manuscript.
